# Identification and Functional Characterization of *Leishmania donovani* Secretory Peroxidase: Delineating Its Role in NRAMP1 Regulation

**DOI:** 10.1371/journal.pone.0053442

**Published:** 2013-01-11

**Authors:** Nisha Singh, Surabhi Bajpai, Vinod Kumar, Jalaj K. Gour, Rakesh K. Singh

**Affiliations:** Molecular Immunology Laboratory, Department of Biochemistry, Faculty of Science, Banaras Hindu University, Varanasi, India; Technion-Israel Institute of Technology, Israel

## Abstract

*Leishmania* silently evades host immune system and establish in the hostile environment of host macrophage phagolysosomes. For differentiation, growth and division parasite acquires divalent cations especially iron from the host nutritive pool. Natural resistance associated with macrophage protein1 (NRAMP1), a cation transporter that effluxes out divalent cations specifically iron from phagosomal milieu to the cytosol, to create ions deprived status for pathogenic microorganisms. The mechanisms of NRAMP1 regulation are largely unknown in leishmanial infections. In the present study, we identified a secretory *Leishmania donovani* peroxidase (Prx) that showed peroxidoxin like peroxidase activity and significantly reduced H_2_O_2_, O_2_.^−^ and NO levels in LPS activated macrophages. Further, we also observed down regulated Nramp1 expression and concomitantly declined labile iron pool in activated macrophages treated with identified peroxidase. Prx also decreased levels of TNF-α, IFN-γ and IL-12 in LPS activated macrophages. These observations indicate a bifunctional protective role of secretory Prx; first it reduces redox activation of macrophages, and secondly it allows iron access to *Leishmania* by down regulating NRAMP1 expression.

## Introduction

Leishmaniases, caused by an obligate intracellular protozoan parasite of the genus *Leishmania*, are endemic in more than 98 countries of tropical and temperate regions [1], [2]. The parasites are carried by 30 species of female sandfly that belongs to the genus *Phlebotamus* in the old world and *Lutzomyia* in the new world [2]. About 20 species of *Leishmania* are responsible for three clinical forms i.e. visceral leishmaniasis (VL), cutaneous leishmaniasis (CL) and mucocutaneous leishmaniasis (MCL). The annual global prevalence of all forms of leishmaniasis is nearly 10 million and approximately 350 million people are at risk. An approximated global burden of VL is about 0.2 to 0.4 million and CL is approximately 0.7 to 1.2 million each year [1], [3], [4]. Most of the VL cases (>90%) occurs in India, Nepal, Bangladesh, Sudan, South Sudan, Ethiopia and Brazil [1], [3]. However, there is a gross under-reporting of the cases from endemic regions and these figures may go up [4]–[6]. In fact, leishmaniasis is occupying pandemic status due to population migration from endemic to non-endemic regions though current statistical data are lacking in disease endemic countries [7].

The *Leishmania* species follow digenetic life cycle; flagellated promastigotes in the sandfly vector and non-flagellated amastigotes in the mammalian host. Soon after the entry into the host, macrophages phagocytose virulent metacyclic promastigotes where they transform into non-motile clinically relevant amastigotes within the phagolysosomes [8]. *Leishmania* adapts two possible strategies for survival within the host macrophages. First, it suppresses macrophages microbicidal activity such as production of superoxide anion (O_2_^−^), hydrogen peroxide (H_2_O_2_), nitrogen species (NOx) and Th1 cytokines [9], [10]. Second, the parasite acquires host nutritive pool specially ions for their growth and survival, which are specifically required for its cellular division and proliferation [11], [12]. In pathogenic protozoans a characteristic defense system is present to protect them from microbicidal free radicals of the host macrophages. This system comprises at least one isoform of superoxide dismutase (SOD) that dismutate superoxide anions to H_2_O_2_. H_2_O_2_ is potentially more toxic than superoxide anion and can diffuse into the parasites more easily but a specific enzyme complex detoxify H_2_O_2_ [13]. *Leishmania* lacks H_2_O_2_ detoxifying catalase and glutathion dependent antioxidant enzymes but expresses enzymes like trypanothione reductase (TR), tryparedoxin (Txn) and tryparedoxin peroxidase (TxnPx)/peroxidoxin (Prx) [14]. These enzymes are mainly responsible for dismutation of host oxidative stress to protect parasitic proteins, DNA and lipids from oxidative damage [15]. The ability of parasite to combat the prooxidants is linked to virulence, pathogensis and resistance in leishmanial infections [16].

*Leishmania* also down regulates the effector function of adaptive immunity, which is characterized by poor cellular immunity and mixed Th1 (IFN-γ, TNF-α, IL-12)/Th2 (IL-4, IL-10) cytokines production [17], [18]. However, the balance is skewed towards Th2 cytokines during active disease [19]. The Th2 cytokines have also been found to be associated with poor peripheral blood mononuclear cells (PBMC) proliferation and macrophage effecter functions during leishmanial pathogenesis [20], [21].

How does *Leishmania* protect itself from hostile environment of macrophages? Still it is unanswered however, it can be rationalized by various strategies followed by parasites in the host. In parsitophorous vacuole of macrophages the parasite faces major challenge of nutrient deprivation especially iron [22]. In addition, iron is also required for their superoxide dismutase activities [13]. To access host iron pool, *Leishmania* species exclusively express a ZIP family iron transporter LIT1 on its surface that transport iron from external environment [22]. However, to counter this survival strategy, host macrophages exclusively recruit natural resistance associated macrophage protein 1 (NRAMP1), a divalent cation pump, on late endosomal/lysosomal compartment that actively efflux out iron from phagosomal milieu to cytosolic compartment [23]. Notwithstanding significant leishmanial research during last few decades either mechanisms of NRAMP1 regulation or parasitic factors that may regulate its function are yet to be identified. This fact drawn our attention that there may be some parasitic specific or default strategies to modulate Nramp1 expression and function to access iron and other cations required for growth, defense and survival. In this study, we report identification, characterization and functional ability of *Leishmania donovani* specific secretory peroxidase (Prx).

## Materials and Methods

### Promastigotes and amastigotes culture

The *Leishmania donovani* (MHOM/IN/80/Dd8) parasites were used in this study. The motile promastigote form of the parasite were cultured in complete Dulbecco's Modified Eagle Medium (DMEM, pH 7.2) containing 10% heat-inactivated fetal bovine serum (FBS), 2 mM L-glutamine, sodium bicarbonate (3.7 gm/L), penicillin (100 U/ml), streptomycin (100 µg/ml) and gentamycin (20 µg/ml) at 26°C in a BOD incubator. DMEM and FBS were purchased from Gibco, USA, and antibiotics were procured from Sigma Chemicals, USA.

Axenic amastigotes were also maintained in the same medium but the medium was acidified to pH 5.5 and incubated at 37°C in humidified CO_2_ incubator containing 5% CO_2_. The amastigotes were characterized by their round or oval shape without flagella and presence of amastigote specific megasomes under phase contrast microscopy. Biochemical characterization was done by lectin agglutination test [24].

### Preparation of leishmanial secretory proteins

For the preparation of secretory proteins both, promastigotes and axenic amastigotes were used. Briefly, parasites were five-six times washed by centrifugation at 3000 rpm for 15 min at 4°C in serum free DMEM to ensure the complete removal of FBS from medium. The washed parasites were suspended at concentration of 1–2×10^8^ parasites per ml in DMEM media without FBS supplemented with antibiotics and incubated under normal promastigote/amastigote culture conditions. During the period of incubation, parasites viability and integrity were assessed by the trypan blue dye exclusion test. The parasites lysis was confirmed by detection of α-tubulin that was detected in culture medium after 72 h. However, 48 h culture medium was used to isolate secretory proteins of promastigotes and amastigotes. In brief, culture medium was centrifuged at 3000 rpm for 20 min at 4°C to pellet down the parasites. The supernatant containing secretory proteins was collected and filtered through 0.22 µm filter unit (Millipore, USA) to remove parasites, if any. After addition of protease inhibitor cocktail (Sigma Chemicals, USA), the supernatant was concentrated about 200–300 folds by ultrafiltration using a 3 kDa-cutoff filter device (Amicon Ultra, Millipore, USA). The concentration of proteins in the supernatant was determined by Lowry's method [25] and stored at −80°C for further use.

### Identification of peroxidase by in-gel peroxidase staining

To identify peroxidase in secretome native polyacrylamide gel electrophoresis was performed according to Laemmli's procedure [26] for in gel peroxidase activity. Proteins (40–60 µg/well, pre-incubated with 0.2 mM dithioerythritol for 30 min at RT) were separated on 10% resolving gel. For detection of peroxidase, the gel was incubated in 4.5 mM guaiacol and 22.5 mM H_2_O_2_ in 100 mM phosphate buffer (pH 6) at 37°C until appearance of the enzyme band. For the determination of relative molecular weight of identified peroxidase, proteins were resolved on 12% SDS-PAGE and bands were visualized by silver staining according to standard protocol. Briefly, gel was first incubated in fixative solution (40% methanol, 10% glacial acetic acid, 50% MillQ water) for 45 min at RT and then incubated in 0.2% sodium thiosulphate with sodium acetate overnight at 4°C. Following day gel was incubated in 0.1% silver nitrate solution for 1 hr at RT. Bands were developed by 3% sodium carbonate and reaction was stopped by 50 mM EDTA. Known molecular weight markers were ran in parallel for densitometric analysis on gel documentation system (Alpha Imager, Alpha Innotech Corporation, USA).

### Recovery of peroxidase from the gel

After peroxidase activity staining, the unstained portion of gel corresponding to the protein was electro-eluted using G-capsule (catalogue number 786-001, G Biosciences, USA) as per manufacturer's instruction. The eluted protein samples were pooled and final concentration was estimated by Lowry's method and stored at −80°C till further use.

### Sequence analysis (LC-MS/MS)

The 25 kDa polypeptide was processed for LC-MS/MS analysis. This facility was availed from the National Institute for Plant Genome Research (NIPGR), New Delhi, India. The obtained sequences were BLAST using NCBI database (http://blast.ncbi.nlm.nih.gov/Blast.cgi) to search its homology. Finally, the alignment of peptide sequence was done using Clustal-W by BioEdit (version 7.0.9.0) software.

### Formulation of peroxidoxin coated latex beads

Latex beads (1.1 µM microsphere, LB-11, Sigma Chemicals, USA) were washed in PBS by centrifugation at 15,000 rpm for 10 min. After washing, beads were suspended in 1 ml PBS containing Prx (at a concentration of 1 mg/ml) and incubated for 24 h at 4°C with continuous mild vortexing followed by centrifugation to remove supernatant. Finally beads were suspended in PBS and stored at 4°C till further use (stable for 1 week).

### Peroxidase activity assay in Prx coated beads

Peroxidase activity of coated beads was analyzed as described elsewhere [16]. In brief, protein content of Prx coated beads and Prx was estimated. Proteins, at a concentration of 0.5 mg/ml, were incubated with 0.2 mM DTE for 30 minutes at RT. For peroxidase assay, Prx and Prx coated beads were mixed separately in a reaction mixture containing 50 mM Tris-HCl (pH 6.0), 0.2 mM dithioerythritol (DTE) and 50 µM H_2_O_2_ and incubated for 5 min. The reaction was stopped after 5 min by the addition of 1 ml of trichloroacetic acid (10%). Further, 0.2 ml of 10 mM ferrous ammonium sulfate and 0.1 ml of 2.5 M potassium thiocyanate was added to the reaction mixture to develop color. The peroxidase activity was determined spectrophotometerically at 480 nm. For calculation of peroxidase activity, a standard graph was plotted ranging from 1 to 50 µM of H_2_O_2_. Peroxidase activity was expressed in terms of amount of H_2_O_2_ consumed (µM/5 Min).

### Isolation and culture of macrophages

Mice peritoneal macrophages were used for this study. Female Swiss albino mice (average wt, 25±5 g) were obtained from the central animal facility of Institute of Medical Sciences, Banaras Hindu University, Varanasi. This study was conducted following Principles of laboratory animal care guidelines (NIH publication number 85–23, revised 1985) and approved by institutional animal ethical committee. Mice were injected (intra peritoneal) with 1 ml of 2% starch solution to activate resident peritoneal macrophages. After two days, 5 ml cold incomplete RPMI-1640 medium with heparin was injected into the peritoneal cavity and cells were drained. The cells were pelleted by centrifugation at 2000 rpm for 5 min followed by washing twice with PBS. Pellets were finally suspended in 1 ml complete RPMI-1640 medium. Cells (10^6^cells/ml) were cultured in 24 well tissue culture plates at 37°C in 5% CO_2_ in humidified condition in CO_2_ incubator (Thermo Scientific, USA). After 2 h incubation, the non-adherent cells were removed by washing with cold serum free medium and adherent cells were further equilibrated for 5 minutes with chilled medium in order to synchronize uptake of beads. Cellular viability of macrophages was determined by trypan blue dye exclusion method.

For execution of experiments, 3 experimental group were made as follows; 1>B, cells activated with only latex beads 2>LPS+B, cells activated with LPS and latex beads and 3>LPS+Prx, cells activated with LPS and peroxidase coated latex beads. LPS (100 ng/ml) was used to activate macrophages except control. The quantity of proteins on latex beads was estimated and beads equivalent to 20 µg/ml of protein were used to treat macrophages wherever required. The non coated latex beads were challenged in the ratio of 20 µl beads per 10^6^ macrophages. The non up taken beads were removed by washing and uptake of beads was confirmed by phase contrast microscopy. Cells were further incubated in the respective experimental conditions for the desired period of experiments.

### Measurement of superoxide anion (O_2_^−^) levels

The super oxide anion content was estimated by the method described elsewhere [27]. The principle of this method is based on the reduction of ferricytochrome c into ferrocytochrome c in presence of O_2_^−^ that directly correlates the level of O_2_^−^ production to the cytochrome c reduced. In brief, 2 ml reaction mixture contained 100 µl of culture supernatant and 0.05 mM ferricytochrom c in PBS. Reaction mixture was incubated for 15 min at 37°C and reactions were terminated by placing the tubes on ice. Absorbance of the supernatant fractions was measured at 550 nm. The concentration of cytochrome c reduced was determined using extinction coefficient 2.1×10^4^ M^−1^ cm^−1^ and expressed as nmoles of O_2_^−^ liberated per mg of protein. The protein content of the macrophages was determined by Lowry's method.

### Hydrogen peroxide measurement

The production of hydrogen peroxide was measured fluoremetrically according to the method described elsewhere [28]. This method is based on the oxidation of p-hydroxyphenyl acetic acid into fluorescent 2, 2′-dihydroxybiphenyl-5, 5′-diacetate in presence of H_2_O_2_ and horseradish peroxidase (HRP). Briefly, after incubation, cells (10^6^) were pelleted and lysed in minimum amount of test buffer (130 mM NaCl, 4.6 mM KCl, 1.1 mM K_2_HPO_4_, 20 mM HEPES, 5 mM glucose, and 1 mM CaCl_2_, pH 7.4). 100 µl of this lysate was mixed with 900 µl of test buffer containing 10 U/ml HRP, 1 mM p-hydroxyphenyl acetic acid and 100 µM NaN_3_. After 15 min incubation at 37°C, fluorescence was measured at wave lengths 334 nm (excitation) and 425 nm (emission). H_2_O_2_ generated was estimated using standard curve precalibrated with known amount of H_2_O_2_ and represented as nmoles per 10^6^ cells.

### Measurement of total nitric oxide (NO_x_)

Total nitric oxide production was assayed in culture supernatant by the method described elsewhere using Griess reagent [29]. Briefly, 100 µl of culture supernatant was added in 96 well microtitre plate followed by addition of 100 µl of freshly prepared Griess reagent (0.1% N-napthyl ethylenediamine and 1% sulfanilamide in 5% phosphoric acid). 100 µl complete medium was used as blank. Plates were incubated for 15 min at RT. The absorbance was taken at 540 nm. The NaNO_2_ (100-3.125 µM) was used to plot standard curve.

### Phosphatase and PTPAse assay

Macrophages were collected and washed twice with TBS (pH 7.0) by centrifugation. Cells were lysed in PTP lysis buffer (50 mM Tris, pH 7, 0.1 mM EDTA, 0.1 mM EGTA, 0.1% 2-mercaptoethanol, 1% Igepal, 25 µg/ml aprotinin, and 25 µg/ml leupeptin) and kept on ice for 45 min. The lysate was cleared by centrifugation, and protein content was determined. Supernatant containing 20 µg of protein was incubated in phosphatase reaction buffer (50 mM HEPES, pH 7.5, 0.1% 2-mercaptoethanol, 10 mM pNPP) for 30 min and absorbance was recorded at 405 nm [30].

Specific PTP activity was determined by the capacity of protein lysate to dephosphorylate a monophosphorylated phosphotyrosine peptide substrate (TRDIpYETDYYRK). Briefly, 20 µg of protein extracts were incubated in phosphatase reaction buffer [50 mM HEPES, pH 7.5, 0.1% 2-mercaptoethanol, 0.5 mM monophosphorylated phosphotyrosine peptide substrate (TRDIpYETDYYRK] for 15 min at 37°C. The reaction was stopped by the addition of trichloroacetic acid (3% final concentration), and the mixture was centrifuged at 15,000 rpm for 5 min. Aliquots of the supernatant were used to determine the free inorganic phosphate released by the PTPase activity with the malachite green (Sigma Chemical, USA), and absorabance was taken at 620 nm. The phosphatase activities of control beads were considered 100% and for other two groups it was represented as percent (%) phosphatase activities relative to control.

### Measurement of cytokines in cell supernatants

The extracellular cytokines levels for TNF-α, IL-12 and IFN-γ were detected by ELISA MAX™ standard set enzyme-linked immunosorbent assay kit as per manufacture instructions (Biolegend, USA) in culture supernatants. The results were represented in pg of cytokines/ml.

### RT-PCR of cytokines mRNA

Total RNA was extracted from stimulated cells with Tri® reagent (Sigma Chemicals, USA) following manufacturer instructions. Briefly, cells were collected and centrifuged at 5000 rpm at 4°C and pelleted. The supernatant was removed and the cells were washed with PBS so that complete media was removed. The cells were then lysed in 300 µl of Tri® reagent and 120 µl chloroform. The suspension was centrifuged at 10000 rpm at 25°C for 10 min. The upper aqueous layer was recovered and twice amount of isopropanol was added. The mixture was centrifuged again at 10000 rpm at 4°C for 10 min and RNA pellets were collected. Finally, RNA pellets were washed with 70% DEPC ethanol three times to remove the impurities. Total RNA was first digested with RNAse free DNase (Fermantas, Germany) to avoid DNA contamination before use. For cDNA preparation, 1 µg total RNA (kept equal for each amplification subjected for densitometric analysis) was subjected to reverse transcription using 20 U M-MLV reverse transcriptase (Fermantas, Germany), 1× RT buffer, 20 mM dNTPs (New England Biolabs, USA), 20 U RNasin (Fermentas, Germany), 0.1 M DTT with DEPC treated water using 200 ng of random hexamers (Fermentas, Germany).

### cDNA preparation and amplification

The cDNA was subsequently amplified by gene specific PCR. The 25 µl of reaction mixture consisted 2 µl cDNA templates, 1×PCR buffer, 0.5 mM MgCl_2_, 200 µM dNTPs, 1 U Taq DNA polymerase (New England Biolabs, USA) and 3.2 µM mice mRNA specific forward and reverse primers for Sod1, Sod2, iNOS, Nramp1 & cytokines (TNF-α, IFN-γ, IL-12). All primers used in this study are listed in Table 1. The β-actin gene was used as house-keeping control gene. Amplification was performed in a thermal cycler (Labnet, USA) programmed for 30 cycles of denaturation at 94°C for 30 s, annealing at 55°C to 46°C depending on the Tm of the primer for 30 s, and extension at 72°C for 30 s, which were preceded by initial denaturation at 94°C for 2 min. Final extension was for 5 min at 72°C. For few amplification conditions were changed as per requirements. The amplified DNA fragments were separated in a 2% agarose gel, with ethidium bromide and photographed under UV illumination on a gel documentation system (Alpha Imager EP, Alpha Innotech Corporation, USA). The relative mRNA expression levels were analyzed by Image Analysis Software (Alpha View^Tm^, Version 2.0.0.9, Alpha Innotech Corporation, USA), wherever required.

**Table 1:** List of mouse specific primers.

SN	Gene	Forward primer	Reverse primer	Product Size (bp)
1	iNOS2	5′GCAATATAGGCTCATCCAG3′	5′AACTCGCTCCAAGATTCC3′	(253)
2	Nramp1	5′CCTACTGGCTTTCTGGATTAC3′	5′AGTTCTGTTTGAGGTGTTCTGG3′	(245)
3	sod1	5′ATTACAGGATTAACTGAAGG3′	5′ CAATGATGGAATGCTCTC3′	(238)
4	sod2	5′ACAAACCTGAGCCCTAAG3′	5′ CTCCCAGTTGATTACATTCC3′	(327)
5	TNF-α	5′CCGATGGGTTGTACCTTGTC3′	5′CGGACTCCGCAAAGTCTAAG3′	(285)
6	IL-12	5′GGAGGACCCATAAGACTGC3′	5′TTTCCCCTTCTTGGAGGTTT3′	(319)
7	IFN-γ	5′GTGATTGCGGGGTTGTATCT3′	5′GGGACAGCCTGTTACTACCTGA3′	(219)
8	β-actin	5′AACCGCGAGAAGATGACCCAGATCATGTTT 3′	5′AGCAGCCGTGGCCATCTCTTGCTCGAAGTC3′	(350)

### Western blotting of proteins

Expression levels of NRAMP1, iNOS, p38 MAPK, pP38 MAPK, ERK1/2, pERK1/2 were analyzed by western blotting using specific antibodies (Santa Cruz Biotechnology, USA and Cell Signaling Technology). The NRAMP1 and iNOS expressions were study after 24 h and signaling proteins were studied after 2 h of macrophages activation in respective experimental conditions. To develop blots, cells were lysed in minimum amount of cold tris buffer saline (TBS: 20 mM Tris HCl, pH 8.0) containing 0.14 M NaCl, 10% glycerol, 1% NP40, 1 mM phenylmethylsulfonylfloride (PMSF), 1 mM sodiumorthovanadate, 1 µM NaF, aprotinin (40 µg/ml) and leupeptin (20 µg/ml), and kept on ice for 30 min. The lysate was centrifuged at 15000 rpm for 10 min at 4°C. The supernatant was collected and protein content was estimated. The supernatant (40 µg of protein/lane) was resolved on 12% SDS-PAGE and transferred on nitrocellulose membrane (Millipore, USA). After transfer, membranes were blocked in blocking buffer [TBST Buffer (TBS+0.1% Tween20) containing 5% BSA] for 1 h at RT and washed thrice with wash buffer [TBS+0.1%Tween 20]. The membranes were incubated overnight at 4°C with NRAMP1, iNOS, p38 MAPK, pP38 MAPK, ERK1/2 and pERK1/2 specific primary antibodies (dilution 1∶200) followed by washing with TBST buffer thrice for 5 min each. Membranes were further incubated with HRP-conjugated secondary antibody (1∶1000) for 1 h at RT. After incubation membranes were washed with wash buffer thrice and blots were developed with 0.03% DAB/0.01% H_2_O_2_ solution. The β-actin was used as reference to calculate relative band intensity that was analyzed by Image Analysis Software (Alpha View^Tm^, Version 2.0.0.9, Alpha Innotech Corporation, USA). The data were expressed as mean ± standard deviation (SD) of band density ratio of three experiments.

### Assessment of labile iron pool (LIP) of macrophages

The labile iron pool (LIP) is redox-active chelatable iron in cytosolic compartment of the cells, which can be directly determined by various fluorescent iron probes [31], [32]. Calcein-AM (Sigma Chemicals, USA) is a membrane permeable dye and in macrophage cytoplasm it is cleaved by non-specific esterases of macrophages into free florescent calcein and non-fluorescent acetomethoxy group. Calcein immediately binds with chelatable iron ions that result in quenched florescence. Briefly, after 24 h incubation in respective experimental conditions, cells (2×10^6^) were incubated with hemin (Sigma Chemicals, USA) at a concentration of 500 µM for 45 min and washed twice with PBS. After washing cell were scraped and suspended in 2 ml PBS in a polypropylene microfuge tube. Further, calcein-AM at a concentration of 0.125 µM was added to the suspension and incubated for 20 min at RT. After incubation, cells were washed to remove extracellular calcein and re-suspended in 2 ml PBS. Fluorescence was measured at excitation wavelength 488 nm and emission wavelength 517 nm. After 2 min interval, salicylaldehyde isonicotinoyl hydrazone (SIH) was added at a concentration of 200 µM. SIH is a strong iron chelator and has more affinity for iron than calcein. It chelates calcein bound iron and releases free calcein that dequench the florescence. After 5 min of SIH addition fluorescence was again measured. The difference in the fluorescent intensity between before and after addition of SIH was proportional to the amount of LIP. The LIP was expressed in terms of arbitrary units of fluorescent intensity ΔF (AU) = fluorescent intensity after SIH addition - florescent intensity before SIH addition).

### Statistical analysis

The data were analyzed by one way analysis of variance (ANOVA) using SNK (Students-Newmann-Keuls) test by Sigma Stat 3.5 software. The *p*-value less than 0.05 were considered significant. Data were represented as mean ± standard deviation. All the studies were performed in triplicate.

## Results

### Identification and characterization of peroxidase active protein

**Figure 1:**
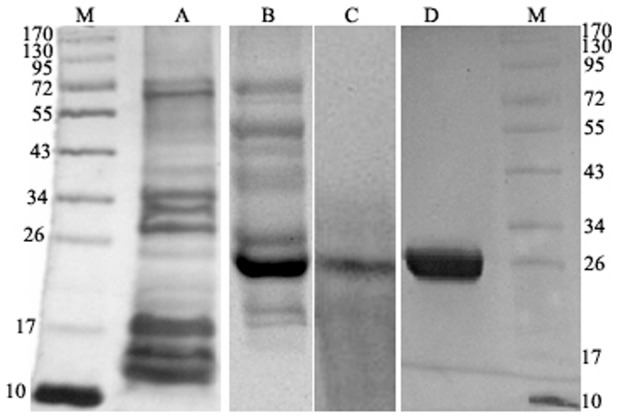
A silver stained gel showing various secretory proteins. Lane **M**: molecular weight markers, **A**: Leishmanial secretory proteins (40 µg/well) on 12% SDS-PAGE ranging from 10–170 kDa, **B**: Secretory proteins on native gel, **C**: Identified peroxidase (25 kDa) on native gel showing peroxidase activity, **D**: Purified 25 kDa protein (20 µg) corresponding to peroxidase on 12% SDS-PAGE (CBB stained gel).

Peroxidase activity staining on native PAGE gel and further elution of protein corresponding to peroxidase active fraction of parasites was subjected to 12% SDS-PAGE showed single band of molecular weight 25 kDa (Fig. 1). Promastigotes secretome showed similar peroxidase active enzyme band however, the level of expression was lower as compared to axenic amastigotes (data not shown). The peroxidase activity of peroxidase in pure Prx and coated beads were found to be 11.2±1.4 and 8.6±0.6 µM of H_2_O_2_ consumed per five minutes, respectively.

### Sequence analysis

**Figure 2:**
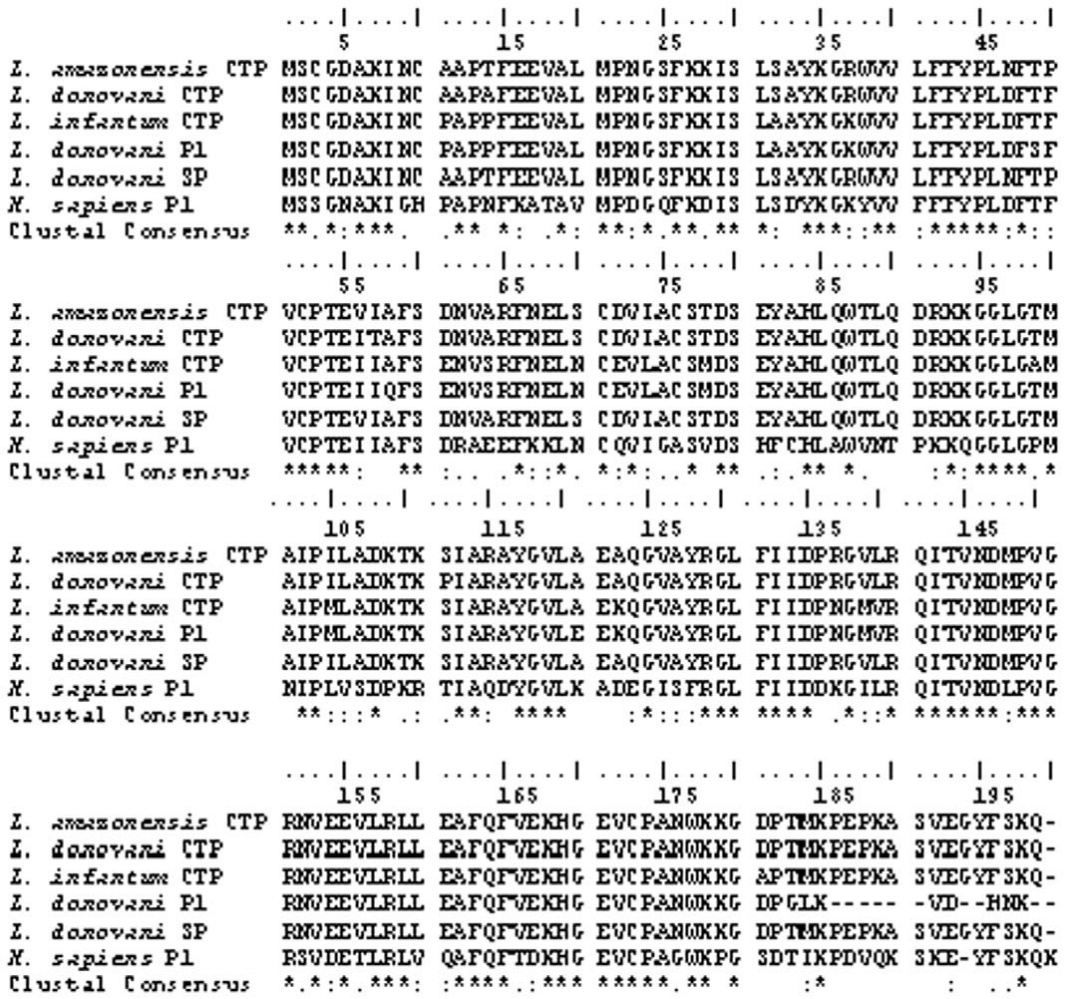
Complete sequence and Clustal W alignment of identified peroxidase. CTP: cytosolic tryparedoxin peroxidase, P1: peroxidoxin1, SP: identified secretory peroxidase (Prx).

The sequence analysis and Clustal W alignment showed 100% homology with *L. amazonensis* cytosolic tryparedoxin peroxidase, 97% homology with *L. donovani* tryparedoxin peroxidase, 90% with *L. infantum* tryparedoxin peroxidase and 88% with *L. donovani* peroxidoxin1 (Fig. 2). The identified protein has two cysteine conserved residues at positions 52 and 173 that are characteristics of peroxidoxin family [16].

### Production of O_2_^−^, H_2_O_2_ and nitric oxide in activated macrophages

**Figure 3:**
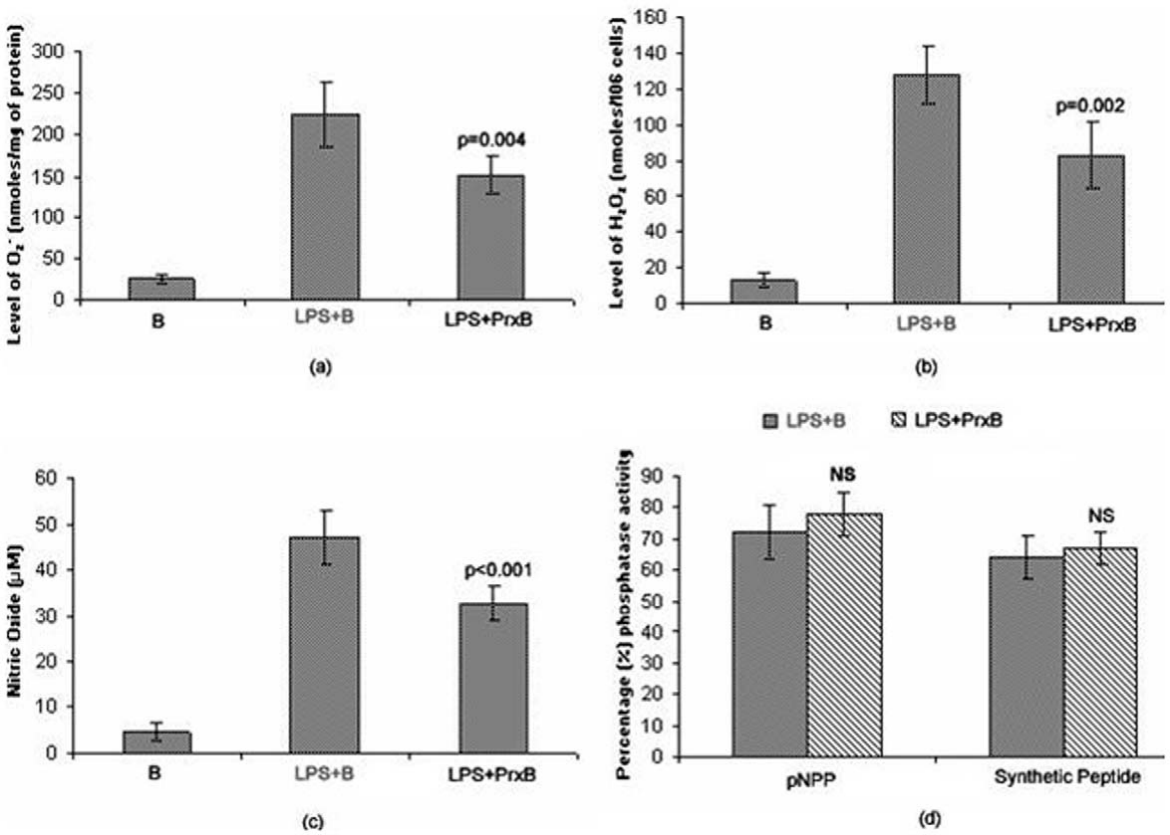
Production of O_2_^−^, H_2_O_2_, NOx and phosphatase activity in various experimental conditions (B, LPS+B, LPS+PrxB). The O_2_^−^, H_2_O_2_ levels and phosphatase activities were estimated after 2 h and NOx was estimated after 24 h in their respective experimental conditions. LPS (100 ng/ml) was used as positive control and control beads have no Prx coating. A significant decrease in the levels of O_2_^−^, H_2_O_2_, NOx was observed in peroxidase treated cells however, total phosphatase and PTPase activities were similar in both groups (student t-test was applied to get significance level between LPS+B and LPS+PrxB).

Respiratory burst activity of macrophages in terms of O_2_^−^, H_2_O_2_ levels was measured after 2 h and NOx levels were measured after 24 h and depicted in Fig. 3a, b and c, respectively. LPS was used as activator of macrophages and treated as positive control in all studies. The levels of O_2_^−^, H_2_O_2_ and NOx in LPS activated cells (LPS+B) were 224±39 nmoles/mg of proteins, 128±16 nmoles/10^6^cells and 47.12±5.76 µM, respectively. In peroxidase treated (LPS+PrxB) cells, their levels were 151±23 nmoles/mg of proteins, 83±19 nmoles/10^6^cells and 32.56±3.9 µM for O_2_, H_2_O_2_ and NOx, respectively. The peroxiadse associated decrease in their levels was significant (p = 0.004 for O_2_^−^, p = 0.002 for H_2_O_2_ and p<0.001 for NOx).

### Total phosphatase and PTPase activity

Since reactive oxygen intermediates inhibit phosphatase activity of cell that eventually prevents dephosphorylation of signaling proteins, we determined total phosphatase and PTPase activities of activated cells after 2 h incubation. Both, total cell phosphatase and specific PTPase activity were determined in macrophage lysates. The cells treated with only beads were used as positive control and their phosphatase activities were considered 100%. The phosphatase and PTPase activities were non-significantly increased in peroxidase treated cells as compared to LPS alone (Fig. 3d).

### Quantization of mRNA expression levels of Nramp1, iNOS, Cu-Zn Sod (Sod1) and Mn Sod (Sod2) genes

**Figure 4:**
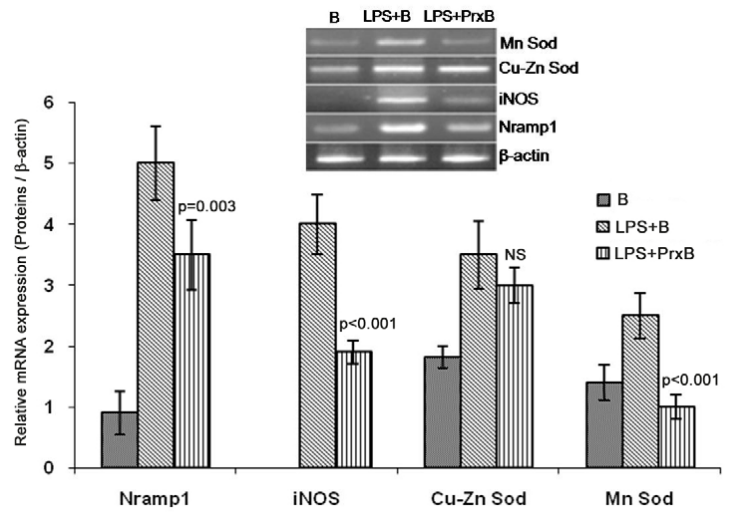
The mRNA expression levels of various proteins involved in macrophage oxidative burst in various experimental groups (B: only beads, LPS+B (beads), LPS+PrxB (peroxidase coated beads). After 24 h of corresponding experimental conditions, total RNA was isolated and amplified using proteins mRNA specific primers (panel A). The relative mRNA expression is represented in the ratio of densitometric values of mRNAs to β-actin. The mRNA levels of all proteins except Cu-Zn Sod were significantly down regulated after Prx treatment (level of significance signifies comparison between LPS+B and LPS+PrxB; NS = not significant).

**Figure 5:**
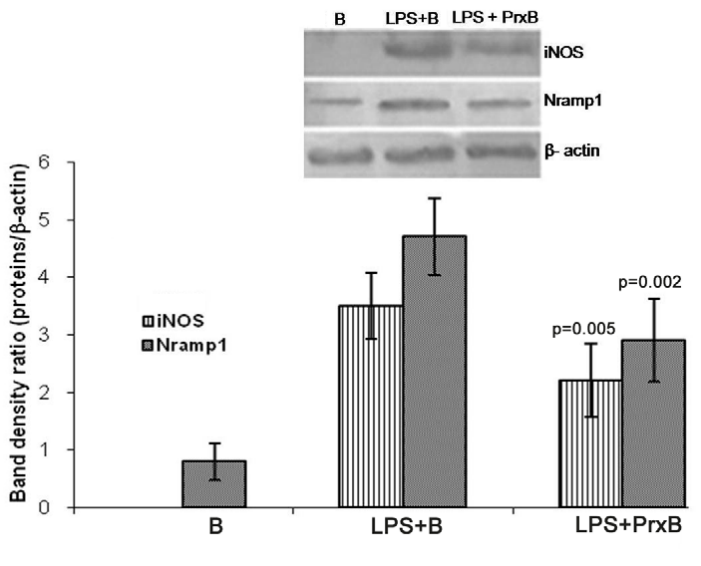
Western blot analysis of iNOS and NRAMP1 in peritoneal macrophages of various experimental groups. Cell lysates were subjected to SDS-PAGE followed by blotting with proteins specific antibodies. Significant decrease in their levels was observed in LPS+PrxB as compared to LPS+B. The upper panel (A) depicts blots showing proteins expression. The lower panel (B) shows densitometric analysis of same with b-actin as control (level of significance is in between LPS+B and LPS+PrxB).

After 24 h incubation in respective experimental conditions, the mRNA expression levels of Nramp1, iNOS, Sod1 and Sod2 in various groups is depicted in Fig. 4. On comparison between LPS+B and LPS+PrxB, the expression levels of Nramp1 (p = 0.003), iNOS (p<0.001) and Sod2 (p<0.001) mRNAs were significantly decreased in peroxidase treated cells. We also estimated NRAMP1 and iNOS expression by western blotting. Prx associated down regulation was significant for both proteins (p = 0.005 for iNOS and p = 0.002 for NRAMP1 (Fig. 5). In control (B) expression of iNOS was not detectable however, NRAMP1 expression was detected.

### Determination of signaling proteins status

**Figure 6:**
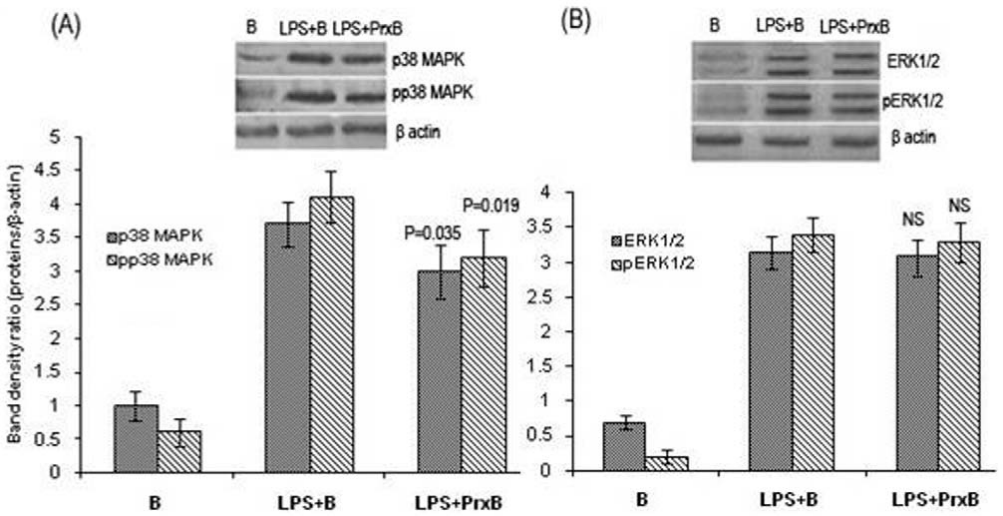
The western blot of signaling proteins involved in LPS induced activation of macrophages. Cell lysates were subjected to SDS-PAGE followed by blotting with proteins specific antibodies. **A:** LPS induced maximum p38MAPK expression and phosphorylation. The peroxidase significantly reduced p38MAPK expression (p<0.035) and phosphorylation status (p<0.019) in macrophages. **B:** ERK1/2 expression and phosphorylation was almost similar (p = NS) in both LPS induced and peroxidase treated macrophages (level of significance is in between LPS+B and LPS+PrxB).

To determine whether secretory peroxidase mediated inhibition of Nramp1 was due to modulated phosphorylation and expression of MAPK's, we further analyzed expression levels of p38MAPK, ERK1/2 and their phosphorylation status (pp38MAPK & pERK1/2). The LPS induced both, expression and phosphorylation of p38MAPK and ERK1/2 as compared to control beads (B). The levels of expression as well as phosphorylation of both proteins were declined in peroxidase treated cells (Fig. 6). However, the levels of p38 MAPK expression (p<0.035) and phoshporylation stauts (p<0.019) was comparatively more declined than ERK1/2 (p = NS for both, phosphorylated and non-phosphorylated) in peroxidase treated cells. These findings suggested that degradation of ROS in macrophages regulates Nramp1 expression through activation and phosphorylation of p38 MAPK not via inhibition of PTPase.

### Production of Th1 responsive cytokines

**Figure 7:**
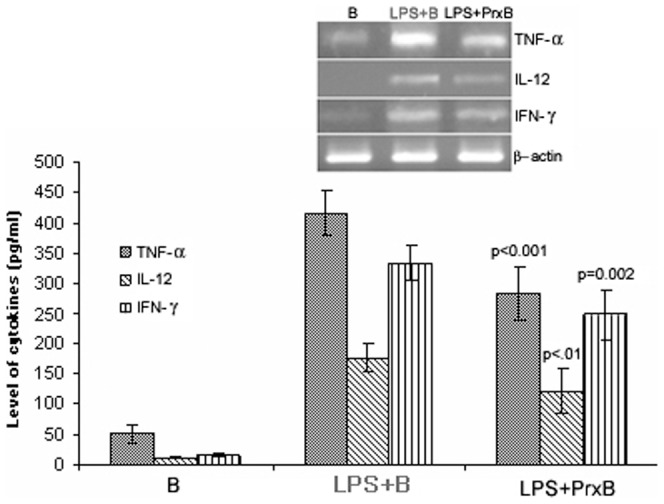
Levels of TNF-α, IFN-γ and IL-12 in cultured supernatant of peritoneal macrophages in various experimental conditions. After 24 h, cell supernatants were collected and cytokines were estimated by cytokine ELISA and values were expressed in pg/ml. The cytokines level was decreased in the peroxidase treated group (LPS+PrxB) as compared to LPS induced cells (LPS+B). The lower picture depicts their relative mRNA expression levels after subsequent amplification with mRNA specific primers (ANOVA was done to obtain significance level between LPS+B and LPS+PrxB).

LPS stimulation resulted in significant (p<0.001) production of all cytokines after 24 h incubation as compared to control beads (B). In LPS induced cells, the levels of TNF-α, IFN-γ and IL-12 were 416.25±37.71, 176.14±24.2 and 334.25±28.41 pg/ml, respectively (Fig. 7). A statistically significant (ANOVA between groups) decrease in the level of all cytokines (TNF-α: 283.81±45.4 pg/ml, IFN-γ: 121.29±37.57 pg/ml, IL-12: 247.5±41.38 pg/ml) was observed in peroxidase (LPS+PrxB) treated cells.

### Labile Iron Pool (LIP) Status of macrophages

**Figure 8:**
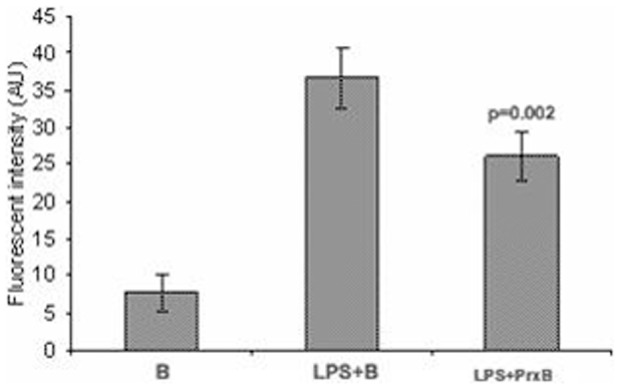
Total labile iron pool of activated cells presented as the difference of fluorescence intensity (ΔF in AU) in stimulated macrophages. The secretory peroxidase decreased LIP status in LPS stimulated cells (comparison between LPS+B and LPS+PrxB).

After 24 h incubation in the respective experimental conditions, the labile iron pool (LIP) in macrophases was determined with calcein-AM. We supplemented macrophages with hemin as a iron source and in all experimental group and LIP was estimated in terms of differential flourescent intensity (ΔF) of calcein, which corresponded to total labile iron pool in macrophage cytoplasm (Fig. 8). The LIP status was lower (p<0.003) in peroxidase treated cells as compared to the LPS activated macrophages.

## Discussion

The present study demonstrates one of the probable survival strategies of *Leishmania* species in the host macrophages. The secretion of peroxidases in host macrophage milieu not only counters oxidative assault but also indirectly responsible for active iron acquisition via down regulation of NRAMP1 divalent cation efflux pump expression and function. In addition, this study also confirms a regulatory role of oxidative and nitrosative stress in leishmanial pathogenesis. We identified and characterized *L. donovani* specific peroxidase in the secretome of both, promastigotes and amastigotes that specifically counters oxidative stress and non-specifically regulates divalent cation transporter (NRAMP1) of host macrophages. To the best of our knowledge, this is the first study that reports a secretory *L. donovani* peroxidase and its role in controlling host macrophages oxidative stress and NRAMP1 function.

Macrophage arsenal is full of its potent armatures such as reactive oxygen and nitrogen species, lysozymes, and other microbicidal molecules to eliminate pathogenic microorganisms [33]. Parasitic survival and proliferation within parasitophorous vacuole primarily depends on its strategy to evade macrophage effecter molecules, diverting immune response and fulfilling its nutritional requirement [10], [11]. However, to flourish within the hostile conditions of the macrophage phagolysosomes, *Leishmania* effectively exploits host immune response to target itself for engulfment into macrophages [34]. Once phagocytosed, the parasite divides and multiplies in the harsh environment of macrophages through inhibition of its effector functions [35].

In leishmanial infection reactive oxygen and nitrogen species play crucial role in initial events of parasite killing and elimination from host. However, during leishmanial pathogenesis their levels decline significantly either by amastigote suppressive mechanisms or by autoredox buffering of host macrophages, which eventually result in diseases onset and progression [36]. LPS is a potent stimulator of macrophages leading to the induction of various inflammatory mediators like reactive oxygen, nitrogen species and inflammatory cytokines production. The identified peroxidase effectively reduced H_2_O_2_ level in LPS activated cells. In addition, we also observed significantly decreased levels of superoxide anions, nitric oxide and Sod genes, which apparently provides an explanatory basis that secretory peroxidases control autoredox buffering of macrophages for parasitic survival inside the hostile environment of macrophages. In both stages of life cycle parasite secretes some active enzymes or factors such as cystein peptidases, acid phosphatases, elF1α, which control various signaling mechanisms that are often beneficial for its survival within the host. Secretory cysteine peptidases activates mitogen activated protein kinases (MAPK) and cleaves NF-κB subunits while elF1α, secreted specifically by amastigotes, binds and activates host SHP-1, which further activates JAK2 and other downstream signaling cascades and eventually control production of various cytokines to facilitate parasite survival [37], [38]. These findings confirmed that secretory Prx complements other peroxidases such as cytosolic and mitochondrial peroxidoxins from oxidants attack for parasite survival [39], [40].

The purpose of host oxidative assault is to not only eliminate parasitic intrusion but also it is a critical link for various inflammatory responses of the host [41]. These species modulate various TLR signaling proteins like MAP kinases, ERK1/2 and various transcription factors such as NF-κB, AP1, which eventually activates inflammatory cytokines production [42]–[44]. H_2_O_2_ is not only a potent oxidizing agent but also act as a secondary messenger and regulates redox signaling through activation of NF-κB pathway and ultimately downstream products such as inflammatory cytokines [45]. The redox status of macrophages also controls nitrosative response and increases production of NOx via iNOS activation. We also observed low levels of NOx and iNOS gene expression in peroxidase treated cells, which may be due to the non-specific decrease in the redox status of host macrophages by secretory peroxidase. Since decreased H_2_O_2_ level eventually augments parasite survival and growth in host macrophages it is quite likely that *Leishmania* effectively uses its secretory peroxidase to silent non-infected macrophages to get an easy entry and survival.

Iron acquisition and utilization is critical for parasitic survival and pathogenesis. It has been observed that any defect in parasitic ability to use and scavenge iron leads to avirulence [11]. How does *Leishmania* modulate iron handling properties of their host cells? It is not well understood yet. NRAMP1 is exclusively expressed on surface of late endosomes/lysosomes and professional phagocytes. The main function of NRAMP1 is to create Fe^2+^, Mn^2+^, Zn^2+^ deprived environment inside the phagosomes by pumping them out from phagosomal milieu to cytosolic compartment of macrophages [23]. Expression of Nramp1 is critical for host resistance as it creates iron deprived condition in phagolysosomes, which is essentially required for intracellular survival of the parasites [46], [47]. In addition, Nramp1 expression also regulates Th1 cytokines production and declined expression of Nramp1 also augment decreased production of TNF-α, IFN-γ, IL-12 [48]. We observed decreased expression levels of Nramp1 in peroxidase treated cell as compared to LPS stimulated cells. LPS positively regulate Nramp1 expression in macrophages [49]. Although, Prx does not exert direct effect on Nramp1 expression and function but reactive oxygen species have been found to regulate its expression via tyrosine and MAP kinase pathways and declined redox status of cell results in decreased expression [50], [51]. These results also acknowledge that redox status effectively controls NRAMP1 status in activated macrophages.

Cellular phosphatases play significant role in signaling mechanisms as they control the phosphorylation status of signaling proteins. Reactive oxygen species inhibit PTPase activity in reversible manner and promotes phosphorylation of signal transduction proteins involved in activation of macrophage effector function [52]. We observed increased phosphatase and PTPase activities in peroxidase treated cells though it was statistically not significant. In this study, secretory peroxidase mediated decline in expression and phosphorylation p38MAPK was more as compared to ERK1/2. This finding suggests that Nramp1 expression is exclusively regulated by p38MAPK [49]. The declined Nramp1 expression was further correlated to decreased LIP status of cells as observed in peroxidase treated cells. The Nramp1 expression is directly related to iron uptake, which is responsible for increased LIP because of the accelerated efflux of iron from phagolysomal milieu to the cell cytosol [53]. These findings suggest that redox status of cell is not only control inflammatory status of activated macrophages but also regulate Nramp1 expression albeit it requires further elucidation of mechanisms.

This study concludes that secretory peroxidase of *Leishmania* effectively reduces host oxidative and nitrosative assault as well as NRAMP1 function, and provides direct protection to the pathogen in the hostile environment of macrophages. It was also evident that the production of Th1 cytokines is controlled by host oxidative stress, in general and parasitic peroxidases, in particular. Since redox status of macrophages direct future course of immune response hence this study provides a basis that an initial control on host oxidative burst direct immune response in the favour of *Leishmania* parasites for their survival. However, a detailed study is required to understand the regulatory role of leishmanial peroxidase as well as to devise a strategy to target peroxidases of leishmanial species for better and effective control of leishmanial infections worldwide.
